# Functional Variation of Two Novel Cellulases, *Pv-eng*-5 and *Pv-eng*-8, and the Heat Shock 90 Gene, *Pv-hsp*-90, in *Pratylenchus vulnus* and Their Expression in Response to Different Temperature Stress

**DOI:** 10.3390/ijms20010107

**Published:** 2018-12-28

**Authors:** Elena Fanelli, Alberto Troccoli, Francesca De Luca

**Affiliations:** Istituto per la Protezione Sostenibile delle Piante (IPSP), SS-Bari, Consiglio Nazionale delle Ricerche, (CNR), 70126 Bari, Italy; elena.fanelli@ipsp.cnr.it (E.F.); alberto.troccoli@ipsp.cnr.it (A.T.)

**Keywords:** cellulases, heat stress, *hsp*-90 gene, oesophageal glands, parasitism, reproduction, RNA interference

## Abstract

Functional characterization of two novel endoglucanase genes, *Pv-eng*-5 and *Pv-eng*-8, of the root-lesion nematode *Pratylenchus vulnus* was carried out. In situ-hybridization experiments revealed that *Pv-eng*-8 transcript was localized in the pharyngeal glands. Silencing of *Pv-eng*-5 and *Pv-eng*-8 resulted in a significant reduction of expression level (52% and 67%, respectively). Furthermore, the silencing of *Pv-eng*-8 determined a reduction (41%) in nematode reproduction, suggesting that treated nematodes are much less able to process food. Surprisingly, no significant difference on reproduction rate was observed with *Pv-eng*-5 dsRNA nematodes, suggesting a neofunctionalization of *Pv-eng*-5 despite the high similarity with nematode endoglucanases. *Pratylenchus* species are poikilothermic organisms showing close relationships with the environmental temperature. The effects of different temperature ranges revealed that the reproductive potential of *P. vulnus* increased with increasing temperature from 23 °C to 28 °C, but no reproduction was observed at 33 °C. In real time, increasing temperature from 23 °C to 28 °C the heat shock gene *Pv-hsp*-90 was differentially expressed in adult stages, while the levels of the effector genes *Pv-eng*-1 and *Pv-eng*-8 in females showed no significant differences compared to those observed at 23 °C, only in males *Pv-eng*-8 level decreased (45%). The upregulation of *Pv-hsp*-90 in both adult stages suggests a protective mechanism in order to cope with unfavorable environmental conditions.

## 1. Introduction

Root-lesion nematodes (RLN) belonging to the genus *Pratylenchus* Filipjev 1936 [1] are among the most damaging plant parasitic nematodes for a wide range of horticultural and ornamental plants ranking third, in terms of worldwide economic losses [2,3,4]. As *Pratylenchus* spp. are migratory endoparasites, they can enter and leave roots, coping both with soil environment, temperature, and plant species [5,6]. Although all plant species contain the same sets of polysaccharides, their relative amounts and layout within the cell wall differ depending on the plant species, cell type, and position and phase of growth and differentiation. Thus, *Pratylenchus* spp. that do not have specialized feeding sites, need to secrete enzymes able to degrade cell wall polysaccharides and soften the walls, enabling the nematodes to feed, migrate, and at last to suppress host defenses. Cell wall-modifying enzymes (CWMPs) have been isolated and characterized from the nematode genera *Heterodera*, *Meloidogyne*, *Pratylenchus*, *Ditylenchus*, *Bursaphelenchus*, *Rotylenchus*, and *Xiphinema* [7,8,9,10,11,12,13,14,15,16,17,18]. The numbers and types of cellulases produced were found to vary among different nematode species [8,9,13,14,19,20]. These cellulases, except for those found in *B. xylophilus* and *X. index*, belong to the glycosyl hydrolase family 5 (GHF5) of β-1,4-endoglucanases and comprise two main categories: catalytic domain with and without a cellulose binding domain.

Soil and temperature impact the reproduction and damage caused by plant parasitic nematodes [21,22,23,24,25,26]. Nematodes are adapted to particular temperature ranges for feeding, hatching, reproduction, and survival. In particular, *Pratylenchus* spp. can adopt a strategy known as anhydrobiosis that allows the juvenile and adult stages to survive periods without plant hosts or severe dry, cold, or hot conditions [27,28]. During anhydrobiosis, metabolism and life processes are slowed down, but *Pratylenchus* spp. resume again as soon as the environmental conditions return to become favorable. So far, little is known about the effect of temperature on penetration, colonisation and reproduction fitness and gene expression in *P. vulnus*. *Pratylenchus vulnus* is a species parasitizing several fruit crop plants all over the world. In Italy, the nematode has been reported as damaging mainly peach and olive but also walnut, sour orange root-stocks, and ornamental plants [29]. This nematode is widespread in rather young olive orchards in the Apulia and Campania regions [30]. Because of its importance, according to the Italian protocol for the production of certified olive nursery plants, both the soil in which the plants are grown and olive plant roots must be free from *P. vulnus* [31]. The present study had two objectives: the first one was the molecular and functional characterization of two endoglucanases, *Pv-eng*-5 and *Pv-eng*-8, of *P. vulnus*. The second objective addressed the relationships between temperature increases, reproduction, and expression of the stress-tolerance and survival-related genes, heat-shock protein 90 (*Pv-hsp*-90) and *Pv-eng*-1 and *Pv-eng*-8 of *P. vulnus*.

## 2. Results

### 2.1. Characterization of Pv-eng-5 and Pv-eng-8 Endoglucanases

In a previous study, a 900-bp PCR fragment was amplified and sequenced [8], showing identical sequence to the partial *Pv-eng*-5 (JN052051) present in the database. Based on this sequence, 5′/3′ Rapid Amplification cDNA Ends (RACE) experiments produced the full-length cDNA of the *Pv-eng*-5. 3′ RACE testing of the *Pv-eng*-5 fragment produced an extra band that was cloned and sequenced. BLASTX search revealed that this fragment corresponded to the partial *Pv-eng*-8 sequence present in the database. 5′ RACE testing on *Pv-eng*-8 produced the corresponding full-length cDNA. The full-length *Pv-eng*-5 cDNA was 1586 bp long, containing an open reading frame (ORF) of 1452 bp, a 29 bp 5′ Untranslated region (UTR) and a 103-bp 3′ UTR which encompassed two adjacent polyadenylation signals.

The full-length *Pv-eng*-8 cDNA was 1228 bp long, composed of a 978-bp open reading frame, a 75-bp 5′ UTR and 175-bp 3′ UTR containing two polyadenylation signals.

Amplification of *P. vulnus* gDNA, using primers located in the UTR regions for both cDNAs, produced a 2258-bp fragment for *Pv-eng*-5 and a 1281-bp fragment for *Pv-eng*-8.

The *Pv*-Eng-5 ORF encoded a deduced protein of 483 amino acids with an estimated molecular weight of 50 kDa and an isoelectric point (pI) of 4.97, while *Pv*-Eng-8 encoded a deduced protein of 325 amino acids and estimated molecular weight of 35 kDa and pI 8.22. *Pv*-Eng-5 and *Pv*-Eng-8 showed a predicted N-terminal signal peptide for secretion with the predicted protease cleavage site between amino acids 22 and 23 for *Pv*-Eng-5 and between amino acids 19 and 20 for *Pv*-Eng-8. *Pv*-Eng-5 protein contains the catalytic domain, the linker, and the Cabohydrate binding module (CBM), type 2, but no peptide linker and cellulose binding domain were present in *Pv*-Eng-8.

BLASTP search revealed that *Pv*-Eng-5 protein showed 80% identity to *Pcr*-Eng-1 and *Pcr*-Eng-2, 68% to *Rs*-Eng-1 and *Rs*-Eng-1A, 60% to *Rs*-Eng-2, and 51% to *Ditylenchus africanus* Eng-1. *Pv*-Eng-8 protein showed the highest identity with the corresponding endoglucanases without the linker and the CBM of *P. penetrans* Eng-2 (77%), *Pv*-Eng-2 (75%) and *Rs*-Eng-2 (63%). The catalytic domain of *Pv*-Eng-8 showed identity to *Pv*-Eng-1 (79%), *Pc*-Eng-1 and *Pv*-Eng-10 (78%), *Pp*-Eng-1 (75%), *Rs*-Eng-1A (64%) and *Pv*-Eng-5 (56%). The consensus sequence IIYETYNEPL of family 5 glycosyl hydrolases, with little differences, was present in all *Pv*-ENGs [32].

**Figure 1:**
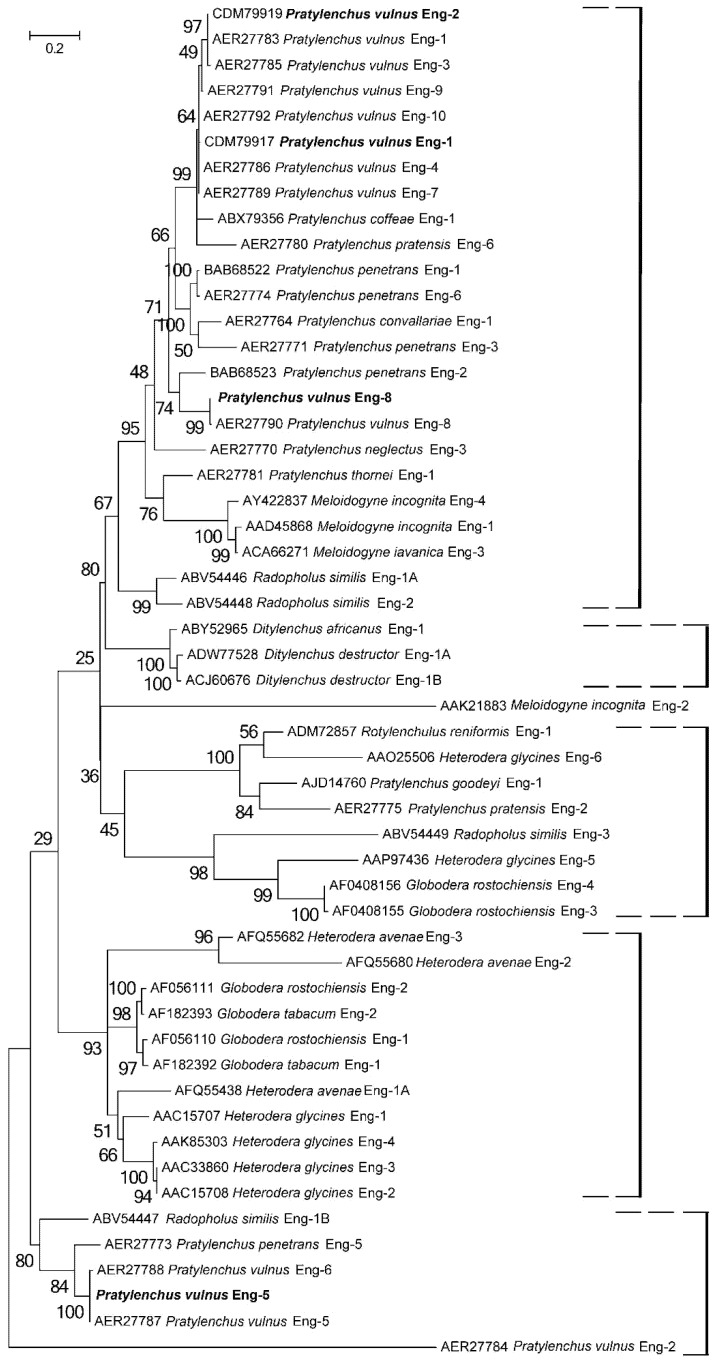
Phylogenetic tree of selected plant parasitic nematode GHF5 cellulase catalytic domains. The Maximum Likelihood method was used to create a bootstrap consensus tree inferred from 1000 replicates.

A phylogenetic tree was generated from the alignment of the catalytic domain of 54 endoglucanase sequences from cyst, root-knot, and lesion nematodes (Figure 1). In the alignment were also included all multiple cellulase genes isolated so far in plant parasitic nematodes. In the Maximum Likelihood (ML) tree, the several *P. vulnus* β-1,4 endoglucanases were grouped in different subgroups with high support. *Pv*-Eng-8 grouped with most of *Pratylenchus* endoglucanases including those of *M. incognita* and *M. javanica* (subgroup I). In contrast, *Pv*-Eng-5 grouped, with 91% support, in subgroup II that included *Pv*-Eng-6, *Pp*-Eng-5 together with several *Globodera*, *Heterodera*, and *Ditylenchus* endoglucanases and *Rs*-Eng-1B. *Rs*-Eng-1A and *Rs*-Eng-2 formed the subgroup III closely related to subgroup I and II. Subgroup IV contained other multiple endoglucanases isolated from different nematode species.

### 2.2. Gene Structure

**Figure 2:**
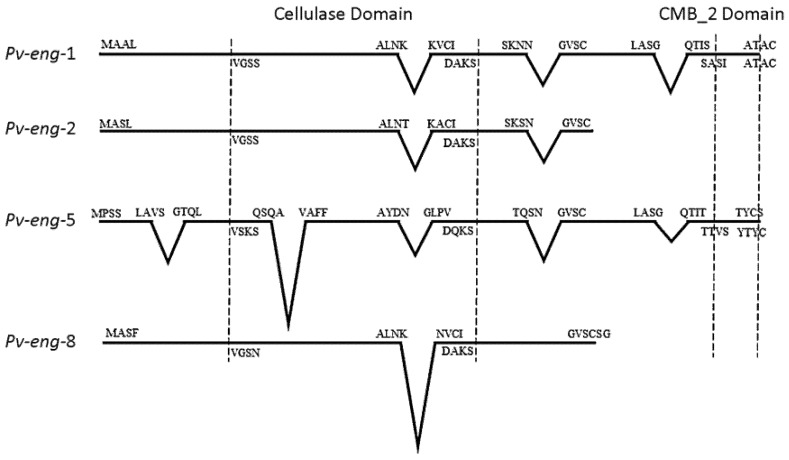
Diagrammatic representation of intron positions among *Pv-eng*-1, *Pv-eng*-2, *Pv-eng*-5, and *Pv-eng*-8 of *Pratylenchus vulnus*.

Alignment of the *Pv-eng*-5 genomic with the corresponding cDNA sequence revealed the presence of six exons and five introns, whilst the *Pv-eng*-8 genomic sequence revealed 2 exons and 1 intron (Figure 2).

The introns in *Pv-eng*-5 were 121, 409, 100, 115 and 61 bp in length, whereas in *Pv-eng*-8, the intron was 303 bp in length. All introns were bordered by canonical *cis*-splicing sequences [33]. The lengths of introns in both *Pv-eng*-5 and *Pv-eng*-8 were larger compared to *Pv-eng*-1 and *Pv-eng*-2 and in general to nematode introns. Introns of *Pv-eng*-5 and *Pv-eng*-8 showed different internal sequences to each other and with *Pv-eng*-1 and *Pv-eng*-2. The last three introns of *Pv-eng*-5 are located in the same conserved positions of the corresponding introns of *Pv-eng*-1 and *Pv-eng*-2 (Figure 2). In addition, the position of the unique intron of *Pv-eng*-8 was conserved in *Pv-eng*-1, *Pv-eng*-2, and *Pv-eng*-5 (Figure 2). Furthermore, the same conserved introns between *Pv-eng*-5 and *Pv-eng*-1 showed the same positions in *P. coffeae Pc-eng*-1. The second intron position of *Pv-eng*-5 is also conserved in *P*. *convallariae Pc-eng*-1, in *Pcr-eng*-1 and *Pcr-eng*-2, in *Pn-eng*-1 and *Pn-eng*-2, in *Pp-eng*-2, *Pp-eng*-3, and *Pp-eng*-4 by comparing the corresponding sequences present in the database [8].

### 2.3. Pv-eng-8 Expression Profile

**Figure 3:**
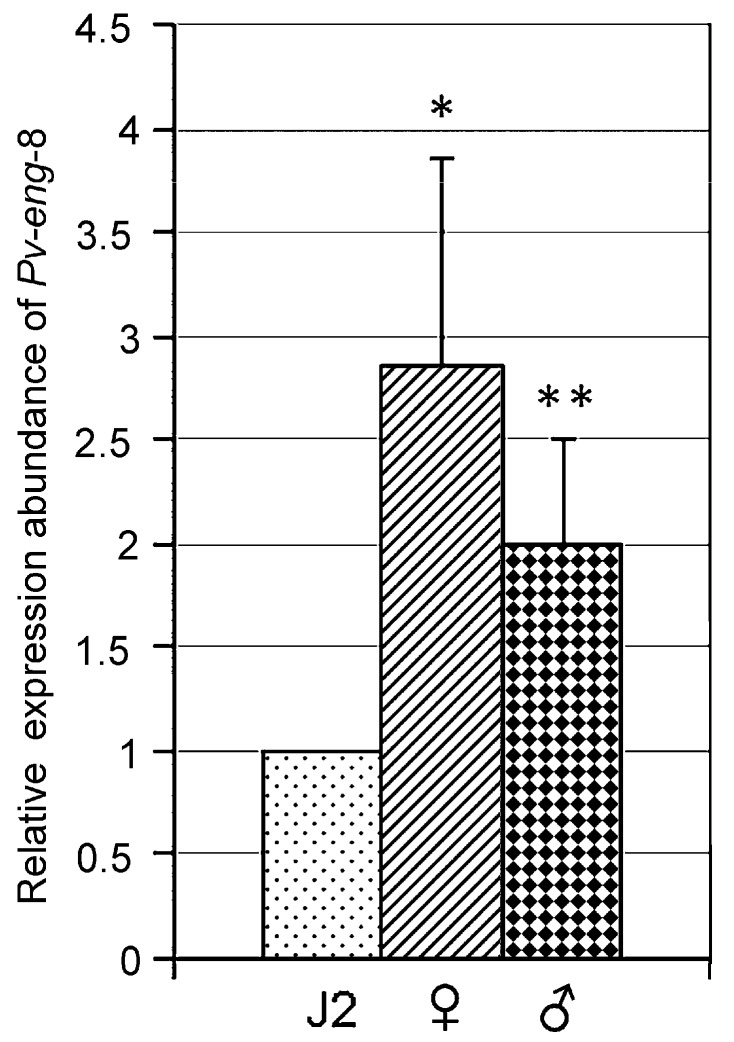
Expression of the endoglucanase *Pv-eng*-8 in juveniles (J2), females and males. Bars indicate standard errors of mean data (*n* = 3). Significant differences were found between the J2 and the adult stages (* *P* < 0.05, ** *P* < 0.01).

The qPCR results showed that *Pv-eng*-8 was expressed in all motile life stages of the nematode (with the exception of eggs and first stage juveniles J1s). The highest levels of *Pv-eng*-8 transcript, relative to second stage juveniles (J2), were observed in adult females (2.8-fold), and males (2.0-fold) (Figure 3).

### 2.4. Tissue Localization of Pv-eng-8 mRNA in P. vulnus

**Figure 4:**
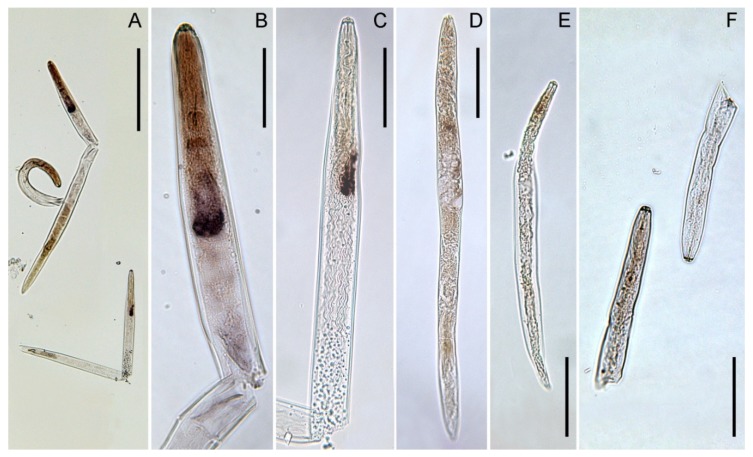
Localization of *Pv-eng*-8 transcript in life stages of *P. vulnus* by in situ hybridization. No staining is observed with the sense probe (**F**). Specific staining of *Pv-eng*-8 antisense probe in females (**A,B**), in males (**C**), and in juveniles (**E**). Slight staining of the *Pv-eng*-8 antisense probe in the gonads of females (**D**). Scale bars: **A** = 200 µm, **B**–**F** = 50 µm.

The *Pv-eng*-8 antisense probe specifically hybridized in the oesophageal gland cells of *P. vulnus* (Figure 4) both in females and males (Figure 4A–C) and resulted in a slight staining even in juveniles (Figure 4E). Sometimes positive staining was observed in the gonads of females (Figure 4D). No hybridization signal was detected in the nematodes when using control sense probes (Figure 4F).

### 2.5. Effects of Pv-eng-5 and Pv-eng-8 Silencing on P. vulnus Reproduction

In order to elucidate the role of *Pv-eng*-5 and *Pv-eng*-8 of *P. vulnus*, RNAi soaking experiments were conducted and the transcript levels were detected by qRT-PCR methods.

**Figure 5:**
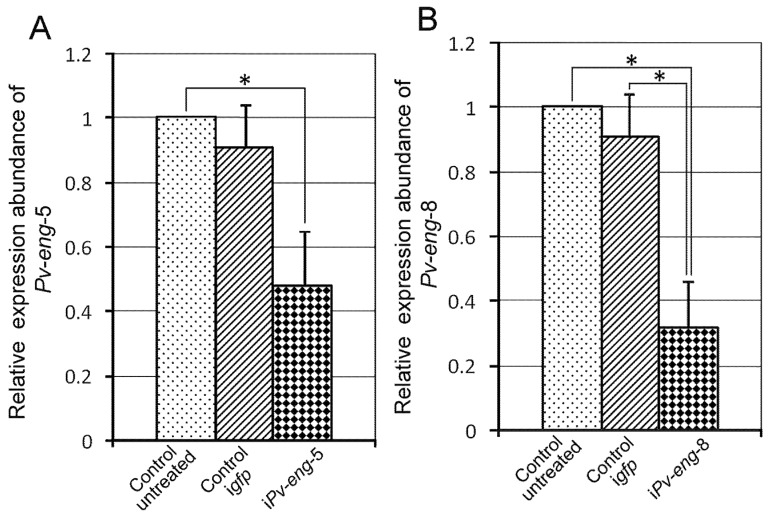
Expression of *Pv-eng*-5 (**A**) and *Pv-eng*-8 (**B**) in *Pratylenchus vulnus* nematodes treated with dsRNA. Controls: expression of *Pv-eng*-5 and *Pv-eng*-8 in untreated nematodes and nematode soaked in *gfp* dsRNA for 24 h. i*Pv-eng*-5: expression of *Pv-eng*-5 in nematodes soaked in *Pv-eng*-5 dsRNA for 24 h (**A**). i*Pv-eng*-8: expression of *Pv-eng*-8 in nematodes soaked in *Pv-eng*-8 dsRNA for 24 h (**B**). Bars indicate standard errors of mean data (*n* = 3). Significant differences (* *P* < 0.05) were found between controls and treated nematodes.

Consistent and statistically significant (*P* < 0.05) reduction in *Pv-eng*-5 and *Pv-eng*-8 expression levels of 52% and 67%, respectively, were observed between dsRNA-treated, untreated, and *gfp* dsRNA-treated nematodes (Figure 5A,B).

To further determine the function of *Pv-eng*-5 and *Pv-eng*-8 in successful parasitism, the corresponding dsRNA-treated nematodes were transferred to mini carrot discs and the ability of nematodes to reproduce was determined. The nematode progenies were counted and compared 19 and 33 days after inoculation.

**Figure 6:**
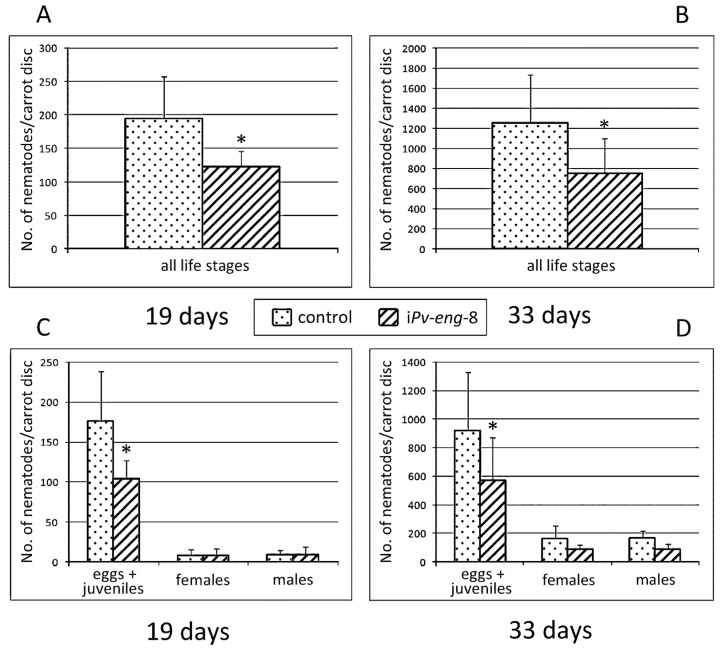
Number of nematodes on carrot discs 19 (**A**,**C**) and 33 (**B**,**D**) days after inoculation of 20 females and 10 males. Control: untreated nematodes; i*Pv-eng*-8: number of treated nematodes by dsRNA for 24 h. Bars indicate standard errors of mean data (*n* = 10). Significant differences (* *P* < 0.05) were found between control and treated nematodes.

The final nematode population recovered from carrot discs inoculated with *Pv-eng*-8 dsRNA-treated nematodes for 19 and 33 days showed a reduction (*P* < 0.05) of 37% and 41%, respectively (Figure 6A,B), compared with that retrieved from discs infected with untreated nematodes. The total number of eggs and larval stages after 19 days was lower in treated nematodes (104) than in control samples (176). No differences were observed in the number of males and females (Figure 6C). After 33 days, the final nematode progeny was still lower compared to control nematodes (Figure 6D). These results indicate that the silencing of *Pv-eng*-8 impairs *P. vulnus* parasitism/invasion and reproduction because treated nematodes are much less able to process food.

**Figure 7:**
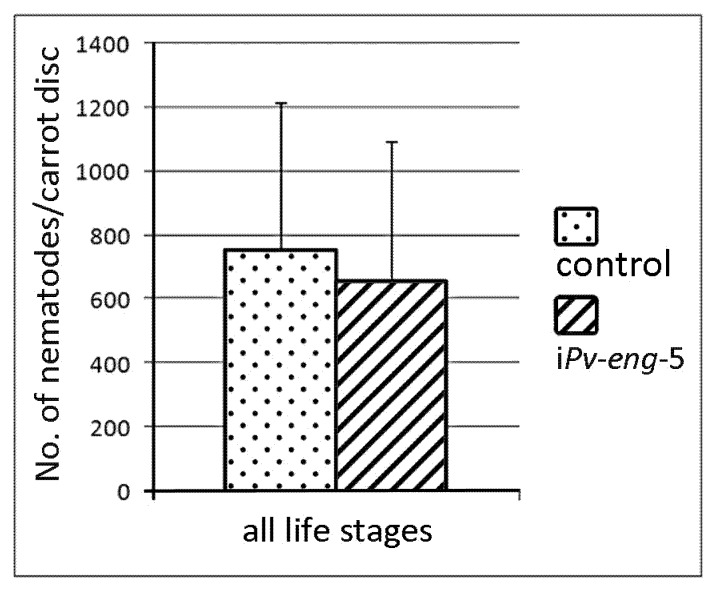
Number of nematodes on carrot discs 33 days after inoculation of 20 females and 10 males. Control: untreated nematodes; i*Pv-eng*-5: number of treated nematodes by dsRNA for 24 h. Bars indicate standard errors of mean data (*n* = 10). No significant differences were found between control and treated nematodes.

Surprisingly, no significant differences on reproduction rate was observed between *Pv-eng*-5 dsRNA-treated and untreated nematodes after 33 days incubation, thus showing a discrepancy with the expression of *Pv-eng*-5 dsRNA-treated nematodes (Figure 7). This result clearly showed that the *Pv-eng*-5 gene is not involved in parasitism.

### 2.6. Effect of Temperature on Reproduction and Motility of P. vulnus

**Figure 8:**
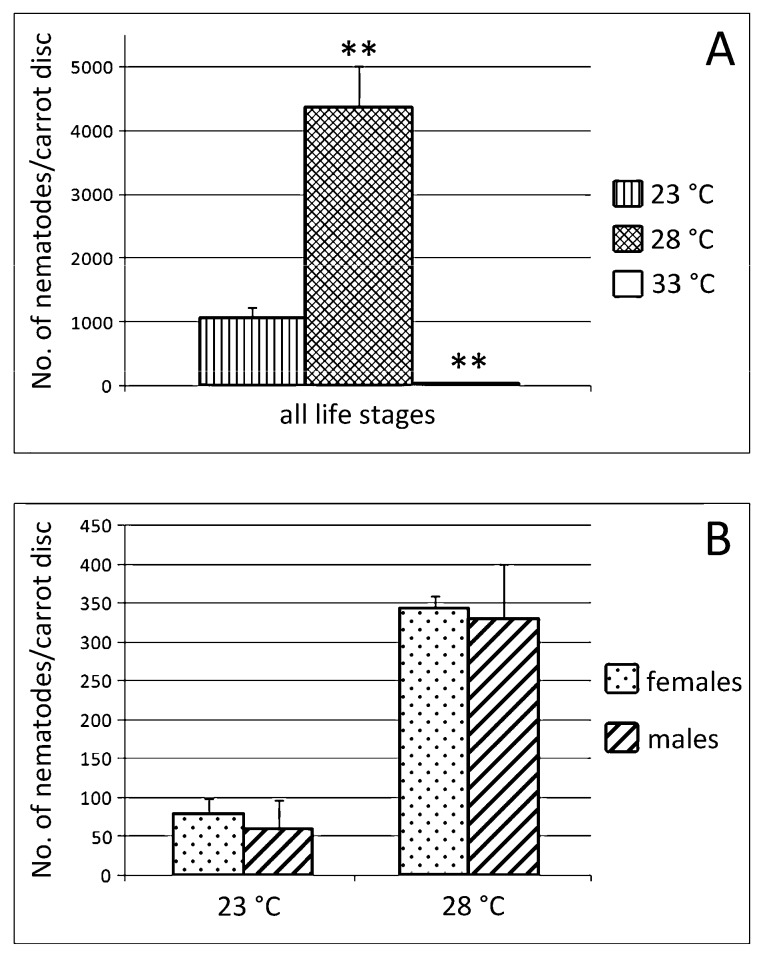
Number of nematodes on carrot discs 33 (**A**,**B**) days after inoculation of 20 females and 10 males at 23, 28, and 33 °C. (**A**): total number of all life stages at 23, 28, and 33 °C; (**B**): sex ratio at 23 and 28 °C. Bars indicate standard errors of mean data (*n* = 10). Significant differences (** *P* < 0.01) were found between control and treated nematodes.

Nematode population densities of Italian *P. vulnus* in relation to incubation temperature after 33 days are presented in Figure 8. The results indicated the greatest reproduction of *P. vulnus* at 28 °C (Reproduction factor (RF) = 145), with the final nematode population increased 4-fold compared to that at 23 °C (Figure 8A). Although the motility was not reduced over a temperature range of 23–28 °C. No differences were found in the sex ratio at 23 and 28 °C (Figure 8B). There was no reproduction at 33 °C (RF = 1) (Figure 8A), suggesting that 33 °C constituted the high temperature stress for *P. vulnus*.

### 2.7. Cloning and Sequencing of the Partial Pv-hsp-90 Gene in P. vulnus

A 354-bp partial fragment of the *Pv*-*hsp*-90 gene was amplified using degenerated primers, and sequenced. Specific primers were designed on the above sequence and used to amplify the corresponding region on the cDNA. The partial *Pv*-*hsp*-90 cDNA and the corresponding genomic sequences revealed the presence of one intron and primers for real time PCR were selected to span the intron.

### 2.8. Expression Profiles of Pv-hsp-90, Pv-eng-1, and Pv-eng-8 under Non Stressed and Stressed Temperature Conditions

Temperature plays a fundamental role in the distribution and abundance of nematodes. Pilot experiments were conducted in order to assess how nematodes respond to different temperatures. Thus, adult males and females were directly exposed at 23, 28, and 33 °C for 2, 4, and 12 h. Increasing the temperature from 23 to 33 °C, *P. vulnus* females exhibited a significant and gradual upregulation of the heat responsive gene, *Pv-hsp*-90 at 2 and 4 h. After longer exposure (12 h) the *Pv-hsp*-90 transcript level dropped to the basal level. On the contrary, *P. vulnus* males showed a strong and significant (*P* < 0.05) increase of the *Pv*-*hsp*-90 transcript after 4 h incubation at 33 °C. After longer exposure, the transcript level dropped almost to basal levels. The transcripts of *Pv-hsp*-90 gene appear to be significantly upregulated in response to temperature increases in females suggesting an adaptive role in maintaining native protein structures from 23 to 33 °C. In males, the highest level of *Pv-hsp*-90 transcript was detected at 33 °C after 4 h incubation, suggesting a lower adaptation of this stage to thermal stress. The parasitism genes, *Pv-eng*-1 and *Pv-eng*-8, showed a little increase in both adult stages from 23 to 33 °C after 4 h incubation, but after longer exposure, the transcript levels dropped to basal level. These data demonstrated that *Pv-eng*-1 and *Pv-eng*-8 transcripts could be quite sensitive at 28 and 33 °C in both adult stages.

### 2.9. Analysis of Cellular Response to Temperature Stress

**Figure 9:**
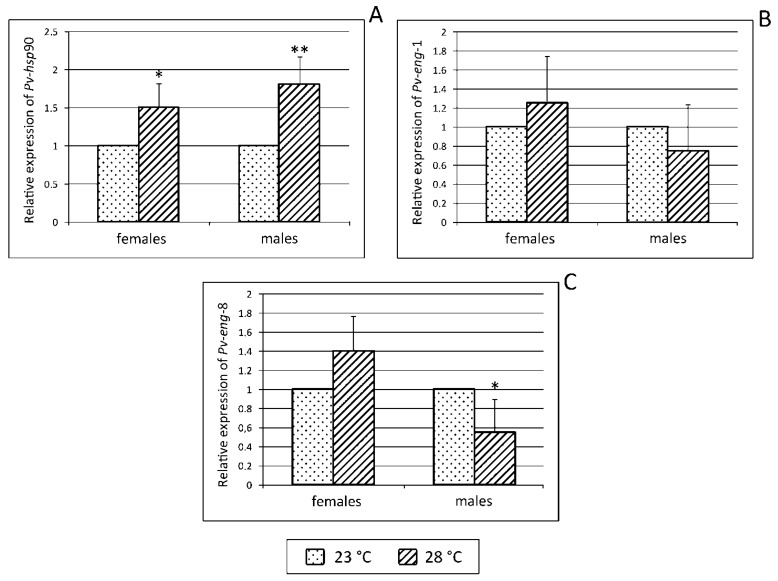
Relative expression of *Pv-hsp*-90, *Pv-eng*-1, and *Pv-eng*-8 (**A**,**B**,**C**, respectively) in females and males 33 days after incubation at 23 and 28 °C. Bars indicate standard errors of mean data (*n* = 10). Significant differences (* *P* < 0.05, ** *P* < 0.01) were found between control and treated nematodes.

To validate the relationships between temperature and nematode migration, reproduction, and survival according to sex, the transcription levels of *Pv*-*hsp*-90, *Pv-eng*-1, and *Pv-eng*-8 were determined in adult males and females recovered from carrot discs incubated at 23 and 28 °C for 33 days. The relative expression of *Pv*-*hsp*-90 both in males and females increased 1.8-fold (*P* < 0.01) and 1.5-fold (*P* < 0.05), respectively, ranging from 23 to 28 °C (Figure 9A). No significant differences were found in *Pv-eng*-1 (Figure 9B) expression in males and females, suggesting that *Pv-eng*-1 might not be sensitive to temperature variation. On the contrary *Pv-eng*-8 expression revealed a significant (*P* < 0.05) reduction (45%) at 28 °C in males; while in females no significant variation was observed (Figure 9C), suggesting that females are more tolerant than males.

## 3. Discussion

Multiple GHF5 B-1,4-endoglucanase genes, showing different structures, have been identified in the root-lesion nematode *P. vulnus* [8,34]. In the present study, we report the characterization of two novel GHF5 endoglucanase genes and the corresponding transcripts, *Pv-eng*-5 and *Pv-eng*-8, from *P. vulnus* in order to increase the understanding of the evolutionary patterns associated with HGT-acquired genes. Both *Pv-eng*-5 and *Pv-eng*-8 endoglucanases had the signal peptide for secretion and a catalytic domain showing low homology with the previously isolated genes in *P. vulnus*. In addition, *Pv-eng*-5 contained the linker and the Carbohydrate binding domain (CBD), while *Pv-eng*-8 contained no linker and CBD. In particular, *Pv*-Eng-8 is the third GHF5 endoglucanase without linker and CBM isolated in *P. vulnus*, confirming the hypothesis that the ancestral gene, after several events of duplication, has undergone sequential losses of linker and CBM [12,20]. It is well known that the acquired genes before separation of the different nematode lineages underwent multiple duplications, thus forming multigene families. Then, after separation, the gene duplication process continued indipendently, leading to novel gene variants with diversification/specialization of function and selective expansion of some genes that are associated with the evolution of the parasitic life style [35,36,37,38,39,40,41]. HGT events of putative cellulases seems to be more frequent in plant parasitic nematodes than in any other trophic groups, but recently it has also been reported in necromenic nematodes belonging to *Pristionchus* genus [42]. The function of these cellulases is still unknown even if they belong to the GH5 family with a different carbohydrate binding module (CBM49 instead of CBM2). It is known that the main roles of CBMs are to increase the performance and the specificity of cellulose hydrolysis, thus the existence of different CBMs of cellulases suggests a central role in the evolutionary development of several cellulolytic enzymes [43]. Based on sequence data, some authors observed that most cellulases lacked CBMs and demonstrated that these cellulases are free in the hydrolysate and could be available for reuse hydrolysing cellulosic and lignocellulosic substrates of epidermal and endodermal cells [43,44].

The identities between catalytic domains of *Pv*-Eng-5 with *Pv*-Eng-8 and *Pv*-Eng-1 were low, 56% and 55%, respectively, while *Pv*-Eng-8 and *Pv*-Eng-1 had 79% identity. These data furtherly support that the *Pv-eng*-5 gene could be associated with a gain of non-canonical activity as also demonstrated by its differential expression during the life cycle and its different tissue localization compared to other *P. vulnus* cellulases. This finding suggests that *Pv-eng*-5 may be involved in plant defense evasion by degrading plant defense compounds [45,46].

Comparison of exon–intron boundaries for all introns among *P. vulnus* endoglucanases showed that most of introns contained the GU-AG type and only *Pv-eng*-8 intron contained the rare GC-AG splice sites [47]. Genome-wide annotation for *Globodera rostochiensis*, *G. pallida*, and *Rotylenchulus reniformis* revealed that the percentages of GC/AG introns were 3.4%, 3.5%, and 2.3%, respectively, the highest reported in nematodes to date [48]. In the free-living nematode *Caenorhabditis elegans*, GC-AG introns have been also found at the same frequency as in humans (0.6% versus 0.7%) and seem to be involved in alternative splicing of developmentally regulated genes [49]. There is a strong conservation of the position and phase of introns among the four complete *P. vulnus* GHF5 genes, as reported in Figure 2, confirming they have evolved from a common ancestral gene. The conservation of some intron positions between *Pratylenchus* engs and *P. vulnus* engs suggests evolutionary conservation rather than parallel gain [8]. Sequence comparisons between introns of *Pv-eng*-1, *Pv-eng*-2, *Pv-eng*-8, and *Pv-eng*-5, located in the same conserved positions, revealed no significant nucleotide similarities confirming old duplications of the ancestral gene and rapid divergence of intron sequences during evolution associated with substantial intron gain rather than intron losses as reported for paralogous gene families [50,51,52]. Gene duplication along with single-nucleotide polymorphisms (SNPs) are major sources of functional diversification because the evolution of paralogs is strongly accelerated as duplication occurs. Phylogenetic analysis based on all GH5 catalytic cellulase domains of plant parasitic nematodes placed *P. vulnus* endoglucanases in three distinct groups (Figure 1), supporting the previous conclusions that in the early Pratylenchidae common ancestor, the HGT event was immediately followed by gene duplications, rapid gene turnover, and sequence diversification [34,36,37,38,39,40,41,53,54]. This evolutionary mechanism is known as subfunctionalization in which each duplicated gene maintains part of functions of the ancestral gene [55].

The expression profile of *Pv-eng*-8 in all life stages with higher level in adult stages, as previously observed for other *P. vulnus* endoglucanase genes [8], highlights the crucial role of this gene together with *Pv-eng*-1 and *Pv-eng*-2 to the successful biotrophic interaction during parasitism of adult stages. In situ localization confirmed that the *Pv-eng*-8 transcript accumulated in the pharingeal gland cells both in females and males. This finding confirms that the pharingeal gland cells of *P. vulnus* remain intact and transcriptionally active in adult stages as observed in other migratory endoparasites such as *D. africanus*, *D. destructor*, and *R. similis* [56,57,58]. It is also remarkable to underline the concomitant expression of all endoglucanases in the adult stages of *P. vulnus*, suggesting the importance of these proteins during parasitism.

To prove further the function of *Pv-eng*-5 and *Pv-eng*-8 in the parasitism, both genes were knocked down by using RNAi and silenced nematodes were incubated on carrot discs for 19 and 33 days. A significant decrease in reproduction (37% and 41%, respectively), compared to the control, was observed only for *Pv-eng*-8 dsRNA. The number of eggs and larval stages as well as adult stages was lower compared to the control, while the ratio between males and females were the same compared to the control. These results confirm that *Pv-eng*-8 gene is involved in migration and feeding of adult stages inside the roots as well as nematode reproduction. Moreover, our results strongly corroborate that the highest expression levels of *P. vulnus* effectors in the adult stage may be related to a less complex association with the host plant during parasitism because they can enter, feed and leave the host root tissues [59], thus suggesting multifaceted functions for this gene family in order to cope with host plants and environment.

In contrast, no difference in the final number of progeny was observed with *Pv-eng*-5 dsRNA compared with the control confirming that this gene does not contribute to fitness of *P. vulnus*. These findings, together with the unique localization of *Pv-eng*-5 at intestine level, demonstrate a direct relationship of the intestine with the environment and *Pv-eng*-5 may be involved in the plant defense evasion and basic defense against environmental toxins while moving inside the roots. Recent studies carried out in *C. elegans, Meloidogyne incognita*, and *Bursaphelenchus xylophilus* showed that there are some putatively secreted proteins, expressed in the intestine, involved in the detoxification of xenobiotic compounds [60,61,62]. These authors suggest that nematodes use a two-layered approach to protect themselves against host cell compounds; initially they secrete some detoxification enzymes into the host, and then others are upregulated in the digestive system or intestinal cells exposed to the ingested plant materials. Altogether, these observations show that in *C. elegans*, *M. incognita*, *B. xylophilus*, and *P. vulnus*, the intestine plays an important role in the evolutionary success of Nematoda in relation to the diverse trophic niches that they occupy [62,63].

In the next decades, significant climate changes, related to temperature increase, are expected and this could result in increased economic damages caused by nematodes all over the world. Studies have also demonstrated that the geographical distribution range of plant pathogenic nematodes may expand with global warming spreading nematode problems to newer areas or crops. Higher temperatures may influence plant pathogenic nematodes directly by interfering with their developmental rate, survival strategies, and indirectly by altering host plant physiology [64].

*Pratylenchus* nematodes are poikilothermic organisms and temperature influences the rates of physiological processes such as movement, growth and reproduction, sex determination, and relative abundance of food and damage to plants [2,65]. To evaluate how higher temperatures influence the rates of physiological processes and reproduction of the Italian *P. vulnus*, we tested different temperature conditions, 23, 28, and 33°C. Our results show that temperature strongly affects reproduction of *P. vulnus* with the greatest population increase (4-fold) at 28 °C compared to that at 23 °C, while at 33 °C no reproduction was observed. These results clearly indicate that *P. vulnus* exhibits adaptation or acclimatization responses at 28 °C, and in contrast, 33 °C constitutes a stress temperature for *P. vulnus*. Little is known about the relationships between survival, temperature, and gene regulation in nematodes. So far, only in two plant parasitic nematodes *M. artiellia* and *B. xylophilous* is it known that exposure to heat stress determines the upregulation of *hsp*-90 gene, involved in thermoregulation and proteostasis [66]. Little is also known about the orthologous gene in RLNs in which thermotaxis plays a central role in migration through the soil, identification of host plants, and migration inside the roots. Thus, the heat responsive gene, *Pv-hsp*-90, and the corresponding transcript of *P. vulnus* were isolated and partially sequenced.

To obtain more insights on thermoregulation in the life cycle of *P. vulnus,* adult-stage nematodes in liquid medium were directly exposed to different temperatures and for different periods of time (2, 4, and 12 h) and the expression profiles of *Pv-hsp*-90 gene were investigated. Our study showed that *Pv-hsp*-90 gene is expressed in both adult stages of *P. vulnus*, thus confirming the importance of this gene in normal growth. By increasing temperature from 23 °C up to 33 °C, *Pv-hsp*-90 gene was upregulated in *P. vulnus* females, suggesting that this stage is significantly heat responsive in order to maintain proteostasis during thermal stress. In males, instead, *Pv-hsp*-90 expression increased after 4 h incubation at 33 °C and, after longer exposure, significantly dropped below the level in the control, suggesting a reduction of the metabolic activity. These in vitro results suggest that adult stages show differential sensitivity to heat stress, in particular females may be the thermo-tolerant stages able to survive higher temperature conditions performing a continuous protective mechanism. To validate the relationships between temperature increase, parasitic behavior, and nematode reproduction, transcript levels of *Pv-hsp*-90 together with two parasitism genes, *Pv-eng*-1 and *Pv-eng*-8, were determined in adult females and males recovered from carrot discs incubated at 28 °C for 33 days. *Pv-hsp*-90 expression was upregulated in both adult stages at 28 °C, confirming that *Pv-hsp*-90 is involved in a continuous protective mechanism against high temperature but within a restricted temperature range (23–28 °C), while in females, *Pv-eng*-1 and *Pv-eng*-8 levels showed no significant differences compared to those observed at 23 °C. Instead, in males, a 45% reduction of *Pv-eng*-8 level was observed (Figure 9) and no significant difference in *Pv-eng*-1 expression occurred. These results, along with those on reproduction, clearly demonstrate that the highest level of *Pv-hsp*-90 at 28 °C in both adult stages play a crucial role as a defense mechanism against high temperature. The expression levels of both parasitism genes do not significantly change at 28 °C in females, while only *Pv-eng*-8 expression decreased in males, confirming that this stage responds differently to temperature increases compared to females. Thus, we can also speculate that the downregulation of *Pv-eng*-8 in males may reflect a slow-down of metabolism of some genes to heat stress in order to ensure reproduction as soon as the environmental conditions become favorable. 

## 4. Materials and Methods

### 4.1. Nematode Collection and Nematode Extraction

An Italian population of *Pratylenchus vulnus* was isolated from olive plant and, starting from single females, reared on sterile carrot discs as described in [8,67].

### 4.2. DNA and RNA Extraction

Total genomic DNA and RNA of *P. vulnus* mixed life stages were extracted using AllPrep DNA/RNA kit (QIAGEN, Madison, WI, USA) according to the manufacturer’s instructions. Genomic DNA and total RNA were quantified using NanoDrop 2000 (Thermo Fisher Scientific, Waltham, MA, USA).

### 4.3. DNA Amplification and Cloning of Pv-eng-*8*, Pv-eng-*5*, and Pv-hsp-*90*

A portion of the *Pv-eng-8* gene was amplified using degenerate primers ENG1/ENG2 [8,13] and the entire gene was obtained by using gene specific primers designed on the corresponding full-length cDNA. The same approach was used to obtain the *Pv-eng*-5 entire gene (Table 1).

The portion of the *Pv*-*hsp*-90 gene was amplified using degenerate primers U831/L1110 (Table 1) [68]. Cycling conditions used were: an initial denaturation at 94 °C for 2 min, followed by 45 cycles of denaturation at 94 °C for 20 s, annealing at 65 °C for 5 s, 60 °C for 5 s, 55 °C for 5 s, 50 °C for 5 s, and extension at 68 °C for 1 min and a final step at 68 °C for 15 min. PCR products were cloned into pGem T-easy vector System II (Promega, Italia, Milano, Italy). Plasmid inserts were sequenced and analyzed. The accession numbers for *P. vulnus* endoglucanases are: LR031566 for the *Pv-eng*-5 gene, LR031565 for *Pv-eng*-5 mRNA, LR031567 for the *Pv-eng*-8 gene, and LR031564 for *Pv-eng*-8 mRNA. The accession numbers for *P. vulnus* HSP-90 are: LR032059 for *Pv-hsp*-90 gene, LR032058 for *Pv-hsp*-90 mRNA.

### 4.4. Rapid Amplification of cDNA ends (RACE)

Race experiments were carried out to obtain the full-length cDNA *of Pv-eng*-5 and *Pv-eng*-8 by using 1 μg of total RNA from nematode mixed stages with SMARTer^®^ RACE 5′/3′ (Clontech, Mountain View, CA, USA) according to the manufacturer’s instructions.

The 3′ end of *Pv-eng*-5 was amplified using the gene specific primer *Pv-eng*-5R3′-2 and a long primer UPM (Table 1). Nested PCR, using the short universal primer UPS and either *Pv*-5DB_for2 or *Pv*-5DB_for3 specific primers (Table 1), generated two bands which were cloned and sequenced. One band corresponded to *Pv-eng*-5 and the second band to *Pv-eng*-8.

The 5′ RACE of *Pv-eng*-8 was generated, using the gene specific primer *Pv-eng*-8REV3 and a long UPM (Table 1). Nested PCR, using the short universal primer UPS and gene specific primers: *Pv-eng*-8-race5′1 and *Pv-eng*-8-race5′2 (Table 1), generated a band which was cloned and sequenced.

### 4.5. Phylogenetic Analysis

Protein sequences of endoglucanase genes were retrieved from the GenBank database according to their accession numbers. Each sequence, including *Pv-eng*-5 and *Pv-eng*-8 from *P. vulnus*, was aligned using MAFFT (EMBL-EBI, Hinxton, UK) [69] implemented in the MEGA package v. 7.2.2 (University of Pennsylvania State, Philadelphia, PA, USA) [70]. The final alignment was checked manually to correct potential inconsistencies. Sequence alignments were manually edited using BioEdit in order to improve the multi-alignment. Outgroup taxa for each dataset were chosen according to the results of previously published data. Phylogenetic trees were performed with Maximum Likelihood (ML) method using MEGA version 7 software [70]. The phylograms were bootstrapped 1000 times to assess the degree of support for the phylogenetic branching indicated by the optimal tree for each method.

### 4.6. Localization of Pv-eng-8 Transcript

In situ hybridization was conducted on all stages of *P. vulnus* using a specific 228-bp fragment of *Pv-eng*-8. Gene-specific forward and reverse primers, *Pv-eng*-8 FOR1/*Pv-eng*-8 REV1 were used to synthesize digoxigenin (DIG)-labeled sense and antisense cDNA probes by asymmetric PCR amplification (Table 1).

The PCR reactions were conducted in a 20-µL reaction mixture with PCR DIG Probe Synthesis kit (Roche Applied Science, Penzberg, Germany). Nematodes were fixed in 2% paraformaldeyde for 18 h at 5 °C, resuspended in 0.2% paraformaldehyde, cut randomly on glass slides with a razor blade and then partially digested with proteinase-K at 22 °C for 30 min [71]. After prehybridization, the sections were hybridized overnight at 50 °C with the denatured sense and antisense probes. Substrate for alkaline phosphatase-conjugated anti-DIG was used to visualize hybridized cDNA probe within nematode specimens using Leitz Diaplan microscope (Wild Leitz, Wetzlar, Germany).

### 4.7. Expression Level of Pv-eng-8

The expression profile of *Pv-eng*-8 transcript was determined in juveniles, females and males of *P. vulnus*. Total RNA was treated with RNase-free DNase I set (Qiagen, Madison, WI, USA). First-strand cDNA was synthesized from 1 µg of total RNA using QuantiTect Reverse transcription kit (Qiagen) following the manufacturer’s instructions.

Real-time PCR was carried out using GO Taq qPCR Master Mix. (Promega) A portion of 18S rRNA gene was used as endogenous control. Gene-specific primers used were *Pv-eng*-8_FOR2/*Pv-eng*-8_REV0 (Table 1).

Relative expression levels were determined using MX3000P software (Stratagene, San Diego, CA, USA) and the 2^−ΔΔ*C*t^ method. All experiments were performed three times on three biological samples.

### 4.8. Pv-eng-8 and Pv-eng-5 Knock-Down by Using ds-RNA

Double-stranded RNA (ds-RNA) was synthesized using MEGA script RNAi kit (Ambion, Thermo Fisher, Waltham, Ma, USA) with the primers T7*eng*5dsfor1/*eng*5dsrev, *eng*5dsfor1/T7 *eng*5dsrev for *Pv-eng*-5 and T7 *Pv*-*eng*-8_FOR2/*Pv-eng*-8_REV0, *Pv*-*eng*-8_FOR2/T7 *Pv-eng*-8_REV0 for *Pv-eng*-8 (Table 1). Non-specific control dsRNA (green fluorescent protein gene, gfp, 250 bp) was amplified by using specific primers GFPFOR/GFPREV from the cloning vector pA7-GFP (Table 1).

Two hundred *P. vulnus* females and 100 males cultivated on carrot discs were collected and soaked either in 40 μL *Pv-eng*-5 or *Pv-eng*-8 dsRNA solution (1 μg/μL) for 24 h. Meanwhile, controls were incubated in either elution buffer or with *gfp* dsRNA. Treated and control nematodes were cleaned three times with DEPC-treated water and total RNA was then extracted. Real-Time PCR was used to analyze transcript suppression after RNAi treatment. All experiments were performed three times.

Gene-specific primers used to analyse *Pv-eng*-8 and *Pv-eng*-5 transcript suppression were *Pv*-*eng*-8_FOR1*/Pv*-*eng*-8_REV1 and *eng*5dsfor2/*eng*5dsrev1, respectively (Table 1).

### 4.9. Effect of Pv-eng-5 and Pv-eng-8 RNAi on Reproduction of the Nematodes

The reproduction tests were conducted on 30 nematodes (20 females and 10 males). Treated and untreated nematodes were inoculated on carrot discs and incubated for 19 and 33 days. The discs were maintained in the dark at 22 ± 1 °C. Each treatment was repeated 10 times. After every incubation time, nematodes were extracted from the carrot discs and mobile stages and eggs were collected.

The sum of the number of eggs, juveniles, females, and males was considered as the final nematode population density (Pf) and was used to determine the reproduction factor.

### 4.10. Effect of Temperature on Reproduction of P. vulnus

Ten replicates of mini carrot discs were each infected with twenty females and ten males and were incubated in three separate incubators set at 23, 28, and 33 °C for 33 days. Preliminary tests indicated that the optimum temperature for the Italian population of *P. vulnus* was 23 °C, thus emphasis was based on choosing incubation temperatures at 5 °C intervals above this temperature. Nematodes were retrieved by cutting the carrot discs into smaller pieces with a scalpel and submerging in water. With a light microscope, the number of eggs, juveniles, females, and males were counted separately. The sum of eggs, juveniles, females, and males was considered the final nematode population density (Pf). The nematode number at different temperatures was calculated.

### 4.11. In Vitro Cellular Response to Different Temperatures

One hundred females and males were incubated separately in a few ml of water for 2, 4, and 12 h at 23, 28, and 33 °C. The nematodes were immediately frozen and total RNA was extracted. The expression profiles of *Pv-eng*-1, *Pv-eng*-8, and *Pv-hsp*-90 were determined by Real-Time PCR. Gene-specific primers used to analyze *Pv-eng*-8 and *Pv-eng*-1 transcripts were: *Pv*-*eng*-8_FOR2/ *Pv-eng*-8_REV0 and LRTfor2/LRTrev, respectively. Gene-specific primers used to analyze *Pv-hsp*-90 were *Pv-hsp*for/*Pv-hsp*rev (Table 1).

### 4.12. In Vivo Cellular Response to Different Temperatures

Infected carrot discs were incubated for 33 days at 23 and 28 °C. One-hundred females and 100 males were quickly collected from carrot discs and frozen in liquid nitrogen. Total RNA was extracted and qPCR was used to determine the transcript level s of *Pv-eng*-1, *Pv-eng*-8, and *Pv-hsp*-90.

## 5. Conclusions

To our knowledge, the present study demonstrates that *P. vulnus* endoglucanases, acquired via HGT, were subjected to several duplications, followed by sequence diversification and the birth of new or specialized function by subfunctionalization, as is the case of *Pv-eng*-5. Furthermore, the present study, for the first time, reports on the fitness and molecular responses to heat stress in *P. vulnus* and on the important roles of *Pv-hsp*-90 and *Pv-eng*-8 genes as protective mechanisms against high temperature.

Finally, the present study reveals that RLN risk is expected to increase in the future as temperature will increase.

## Figures and Tables

**Table 1:** Primers (MWG-Operon) used for cloning and expression analysis of *Pratylenchus vulnus* endoglucanases, *hsp*-90 gene, and *gfp* gene.

Primer	Sequence 5′–3′
ENG1	TA(T/C)GTIAT(T/C/A)GTIGA(T/C)TGGCA
ENG2	GTICC(A/G)TA(T/C)TCIGTIAC(A/G)AA
U831	AAYAARACMAAGCCNTYTGGAC
L1110	TCRCARTTVTCCATGATRAAVAC
*Pv*-5DB_for2	AGTCGAGCGGCGATGGCAC
*Pv*-5DB_for3	GGCACTGTCGACACTTCGG
*Pv-eng*-8_REV3	CTTGGTGCGAAGATCGGCC
*Pv-eng*-8-RACE5′1	CTGCGCAATTGCCGCATTC
*Pv-eng*-8-RACE 5′2	CGCTTGGATTGCTCAAATAGC
*Pv-eng*-8_FOR1	GAACAATCTTCGAAGGACT
*Pv-eng*-8_REV1	ACCCCCTCCCATTGTCTAGCC
*Pv-eng*-8_FOR2	GGCTAGACAATGGGAGGGGGT
*Pv-eng*-8_REV0	GGTCGAATTTATGAGTTAGCGG
T7*eng*5dsfor1	TAATACGACTCACTATAGGGCATCACTAGCTCGTGGAAT
*eng*5dsrev	CAGTATGATGTTCAGGAGCA
*eng*5dsfor1	CATCACTAGCTCGTGGAAT
T7*eng*5dsrev	TAATACGACTCACTATAGGGCAGTATGATGTTCAGGAGCA
T7*eng*8_FOR2	TAATACGACTCACTATAGGGGGCTAGACAATGGGAGGGGT
T7*eng*8_REV0	TAATACGACTCACTATAGGG GGTCGAATTTATGAGTTAGCGG
GFPFOR	CACATGAAGCAGCACGACT
GFPREV	GATATAGACGTTGTGGCTGT
*Pv*-*hsp*-FOR	GGACGCGTAATCCGGATGA
*Pv*-*hsp*-REV	ACGGAGCGCGTTGGGGAAC
LRTfor2	TCTGCGTCCGCATCGATTAC
LRTREV	TGGCTAATCAGCATGCAGTG
UPM	CTAATACGACTCACTATAGGGCAAGCAGTGGTATCAACGCAGAGT
UPS	CTAATACGACTCACTATAGGGC
